# Probiotic Properties of New *Lactobacillus* Strains Intended to Be Used as Feed Additives for Monogastric Animals

**DOI:** 10.1007/s12602-020-09674-3

**Published:** 2020-06-23

**Authors:** Katarzyna Śliżewska, Agnieszka Chlebicz-Wójcik, Adriana Nowak

**Affiliations:** 1 Institute of Fermentation Technology and Microbiology, Faculty of Biotechnology and Food Sciences, Lodz University of Technology, Wólczańska 171/173, 90-924 Łódź, Poland; 2 Department of Environmental Biotechnology, Faculty of Biotechnology and Food Sciences, Lodz University of Technology, Wólczańska 171/173, 90-924 Łódź, Poland

**Keywords:** Probiotic, *Lactobacillus*, Aggregation, Adhesion, Antagonism, Survivability

## Abstract

The study aimed to evaluate the safety and probiotic properties of selected *Lactobacillus* strains, which are intended to be fed to monogastric animals. The *Lactobacillus* spp. appeared to be safe since they did not degrade mucus and did not exhibit β-haemolysis. Moreover, the survival of Caco-2 cells in the presence of metabolites of the selected strains was high, which also indicated their safety. The analysed strains showed moderate or strong antagonistic activity against *Salmonella* spp., *Listeria monocytogenes*, *Campylobacter jejuni* and *Campylobacter coli*, which was tested with the usage of the agar slab method. Furthermore, the strains showed high survivability in an acidic environment and the presence of bile salts (~90%). High resistivity or moderate susceptibility to antibiotics was also observed, as a result of the disc diffusion method. The strains were mostly moderately hydrophilic (hydrophobicity: 10.43–41.14%); nevertheless, their auto-aggregation capability exceeded 50% and their co-aggregation with pathogens varied between 12.12 and 85.45%. The ability of the selected strains to adhere to Caco-2 cells was also analysed; they were found to be moderately adhesive (85.09–95.05%) and able to hinder pathogens attaching to the cells (up to 62.58%). The analysed strains exhibit probiotic properties, such as high survivability and adherence to epithelial cells; therefore, they are suitable for administration to monogastric animals. Since the overuse of antibiotic growth promoters in livestock leads to the spread of antibiotic-resistant pathogens and accumulation of chemotherapeutic residues in food of animal origin, it is of vital importance to introduce alternative feed additives.

## Introduction

The *Lactobacillus* genus consists of Gram-positive, non-spore-forming and facultatively anaerobic or microaerophilic rod-shaped bacteria which belong to the *Lactobacillales* order and constitute one of the lactic-acid-producing bacteria genera (LABs) [1]. LABs are comprised of over 200 species and subspecies of *Lactobacillus* sp., as well as *Lactococcus* sp., *Streptococcus* sp., *Enterococcus* sp., etc., which can be used as probiotics [2–4].

Live microorganisms that can contribute to the improvement of host health, when administered in the proper amounts, are called probiotics [5, 6]. Prior to their in vivo administration, the beneficial functionality and safety of the isolated microorganisms must be assessed [7]. The human gastrointestinal tract (GIT) is considered a safe environment to isolate potentially probiotic microorganisms. Nevertheless, alternative habitats are screened to obtain new beneficial strains [8]. Nowadays, novel probiotics are isolated from various natural sources, such as plants, soil, animals’ GITs and dairy products [9].

One of the most important probiotic properties is the ability to survive passage through the digestive tract, as well as the ability to adhere to the intestinal epithelium, which can allow them to colonize the GIT of the host [4, 10]. Probiotics’ adhesion can be related to their cell surface characteristics, such as hydrophobicity, which if it is high enough can result in strong interaction with mucose. However, not only the hydrophobicity of probiotic cells’ surfaces is responsible for their attachment to epithelial cells but also more specific mechanisms involving lipoteichoic acid, extracellular components (exopolysaccharides or proteins) or surface proteins [11, 12]. Furthermore, the auto-aggregation capacity of probiotic microorganisms, which is defined as the ability of cells of the same kind to self-adhere, is related to their adhesive properties [8, 13]. Auto-aggregations of probiotic cells ensure microorganisms can reach a higher population density and stability in the host GIT, as a result of reduced exposure of the cells to unfavourable conditions [8, 14]. In addition to that, the co-aggregation capability, which is defined as the binding of organisms of diverse species, is considered a vital probiotic feature, since it can prevent the GIT of the host from being colonized by pathogens [13, 15]. The antimicrobial activity of probiotics is also attributed to the competitive exclusion of pathogens through antagonism for the binding site and nutrients, production of inhibitory metabolites against unfavourable microbes and stimulation of the host’s immune system [16].

Pathogenic bacteria, such as *Salmonella enterica* subsp. *enterica* serovar Typhimurium, *Salm. enterica* subsp. *enterica* serovar Choleraesuis or enterotoxigenic *Escherichia coli* strains (ETEC), are often responsible for infections in livestock animals, which can lead to inflammation in their GITs, diarrhoea and even sepsis. Moreover, *Campylobacter* sp. and *Listeria monocytogenes* can reside in GITs of monogastric animals, which can cause infection; however, livestock can also serve as an asymptomatic carrier of these pathogens [17]. These potentially harmful bacteria can be transmitted from animals to humans, causing diseases. Since livestock animals are more susceptible to pathogens, the prevalence of bacteria should be controlled at the farm level [18]. In order to prevent pathogenic bacteria from spreading in animals’ GITs antibiotics can be used, which helps improve carcass quality, ensure economic production and improve breeding efficiency [19]. Due to the widespread issue of bacterial antibiotic resistance and the presence of drug residues in animals, alternatives to antibiotic growth promoters (AGPs) are being sought, for which probiotics are well-established substitutes [20, 21].

Assessment of the probiotic features of selected *Lactobacillus* strains in vitro and their applicability to further in vivo studies in monogastric animals was the main aim of this research.

## Materials and Methods

### Microorganism, Medium and Propagation

Five *Lactobacillus* strains, with desirable probiotic features, were selected from a collection of 53 lactobacilli isolates for the present study. The bacterial strains were isolated with the use of Rogosa Agar (BD Difco™, Detroit, MI, USA) from plant silage (*Lact. plantarum* ŁOCK 0860), caecal content of sow (*Lact. paracasei* ŁOCK 1091) or piglet (*Lact. reuteri* ŁOCK 1092) and form poultry dung: broiler chicken (*Lact. pentosus* ŁOCK 1094) or turkey (*Lact. rhamnosus* ŁOCK 1087). These strains are on deposit in the Lodz Collection of Pure Cultures (ŁOCK) of the Institute of Fermentation Technology and Microbiology (Lodz University of Technology, Lodz, Poland).

Six pathogenic, enteric bacterial strains were also used for the study: *Salmonella enterica* subsp. *enterica* serovar Typhimurium ATCC 13311 (*Salm*. Typhimurium), *Salm. enterica* subsp. *enterica* serovar Enteritidis ATCC 13076 (*Salm*. Enteritidis), *Salm. enterica* subsp. *enterica* serovar Choleraesuis PCM 2565 (*Salm*. Choleraesuis), *Campylobacter jejuni* NCTC 11322, *Camp. coli* PCM 2623 and *Listeria monocytogenes* ATCC 13932. The strains were obtained from the American Type Culture Collection (ATCC; Manassas, VA, USA), the Polish Collection of Microorganisms (PCM; Wroclaw, Poland) and The National Collection of Type Cultures (NCTC; London, UK).

Cryobanks™ (Copan Diagnostics Inc., Murrieta, CA, USA) were used to store the strains at a temperature of −22 °C. Prior to analysis, activation and two passages of the *Lactobacillus* and the pathogenic strains were made in de Man, Rogosa and Sharpe broth (MRS; Merck Millipore, Darmstadt, Germany) or nutrient broth (Merck Millipore), respectively. The strains were incubated at 37 °C for 24 h, without oxygen limitation—with the exception of the *Campylobacter* spp. which were cultivated in anaerobic conditions with the use of anaerostat (Oxoid™ AnaeroJar™; Thermo Fisher Scientific Inc. Waltham, MA, USA) and gaspak (Oxoid™ AnaeroGen™; Thermo Fisher Scientific Inc.).

### Caco-2 Cell Cultivation

Human colon adenocarcinoma cell line (Caco-2) was obtained from the ATCC (lot no. 58844056). The cells were cultured in Roux flasks as monolayers in Dulbecco’s Modified Eagle’s Medium (DMEM, Sigma-Aldrich, St. Louis, MO, USA) supplemented with 10% foetal bovine serum (FBS; Gibco, Thermo Fisher Scientific Inc.), 4 mM of GlutaMAX™ (Gibco, Thermo Fisher Scientific Inc.), 25 mM of HEPES (Sigma-Aldrich), 100 μg/ml of streptomycin and 100 IU/ml of penicillin (Sigma-Aldrich). Caco-2 cells were cultured at 37 °C in 5% CO_2_ for 7–10 days, during which time the adherent cells were washed every 3 days with 0.1 M phosphate-buffered saline (PBS; Calbiochem®, Merck Millipore) and fresh medium was added. Subsequently, after the monolayer was formed, the cells were detached by trypsinization with 1% trypsin-EDTA (Sigma-Aldrich) for 2 min at 37 °C and the cells suspension was centrifuged (187×g, 5 min). Fresh DMEM was added to the biomass; afterwards, the cell count was performed with a haemocytometer; and cell viability was determined by trypan blue exclusion.

### Safety Assessment

#### Haemolytic Activity

Selected *Lactobacillus* strains were analysed for their haemolytic activity on MRS agar with 5% (*v*/v) defibrinated sheep blood (Graso® Biotech, Starogard Gdański, Poland), based on the method previously described by Guerra et al. [22]. Each strain was analysed in two modes of inoculation: streaking and stabbing into the agar, in three repetitions. After incubation at 37 °C for 48 h, the plates were screened for the results of α-haemolysis (grey-green halo) or β-haemolysis (transparent halo).

### Mucin Degradation

A mucin degradation assay was performed in the agar medium B, both without and with 1% (*w*/*v*) glucose, as well as with 0.3% (w/v) mucin from porcine stomach (Sigma-Aldrich), following Zhou et al. [23].

The activated monocultures of *Lactobacillus* strains were inoculated (10 μl) onto the surface of the medium and incubated at 37 °C for 72 h. Afterwards, 0.1% (*v*/v) amido black in 3.5 M acetic acid was used to stained the plates, which after 30 min were washed with 1.2 M acetic acid. Mucin lysis was observed as a discoloured halo around the colony.

### Cytotoxic Activity of Bacterial Culture Supernatants (BCS) Towards Caco-2 Cells

The active monocultures of *Lactobacillus* strains were centrifuged at 10,732×g relative centrifugal force (RCF) for 15 min (Centrifuge MPW-352; MPW, Warsaw, Poland) to obtain the cell-free filtrates. Subsequently, the 0.22 μm syringe filters (Millex-GS, Merck Millipore) were used to filtered supernatants, which were then stored at −22 °C until use.

The cytotoxic potential of the bacterial culture supernatants (BCS) was investigated by using a Neutral Red Uptake (NRU) assay. Caco-2 cells in the complete culture medium were placed in a 96-well plate (Corning Inc., Corning, NY, USA) in the amount of 1 × 10^4^ cells per well and incubated overnight at 37 °C in 5% CO_2_ in order to attach. Afterwards, the medium was removed by aspiration, and the cells were exposed to the BCS at the final concentrations of 5, 10, 20 and 50% (*v*/v), in four repetitions. Caco-2 cells without tested agent were used as a control sample. Incubation of the cells was conducted in a CO_2_ atmosphere at 37 °C for 48 h, then the medium with BCS was removed. Next, to each well 100 μl of neutral red dye (50 μg/ml in PBS; Sigma-Aldrich) was added, and plate was incubated at 37 °C under 5% CO_2_ for 3 h. Thereafter, neutral red was extracted with 50 μl of acidified ethanol solution (1% [*v*/v] acetic acid; 50% [v/v] ethanol; 49% [v/v] distilled water). Microplate reader (TriStar^2^ LB 942, Berthold Technologies GmbH & Co. KG, Bad Wildbad, Germany) was used to measure the absorbance at 550 nm with a reference filter of 620 nm. Cell viability (%) was calculated using the formula provided below:

$$ Cell\ viability\ \left(\%\right)=\left( sample\  OD\times control\  OD\right)\times 100\%, $$

where the control sample constituted 100% Caco-2 cells’ viability. The results obtained were presented as the mean value with standard deviation (SD) included.

### Probiotic Properties Assessment

#### Antagonistic Activity of *Lactobacillus* Spp. Against Pathogenic Bacteria

The antagonistic activity was investigated on MRS agar medium using the agar slab method [24]. The *Lactobacillus* spp. (10^8^ cells/ml) were introduced into MRS agar medium, poured into Petri dishes and incubated at 37 °C for 24 h. Next, 10-mm-diameter slabs (in triplicate) were cut from the solidified MRS medium overgrown with the probiotic strains and applied to the prepared nutrient agar, containing test strains of pathogenic bacteria (10^6^ cells/ml). The plates were incubated at 37 °C for 18 h without oxygen limitation in the case of *Salm.* Choleraesuis PCM 2565, *Salm*. Enteritidis ATCC 13076, *Salm*. Typhimurium ATCC 13311 and *L. monocytogenes* ATCC 13932. Anaerobic conditions were provided, by anaerostat (Oxoid™ AnaeroJar™; ThermoFisher Scientific Inc.) and gaspak (Oxoid™ AnaeroGen™; Thermo Fisher Scientific Inc.), for the cultivation of *Camp. jejuni* NCTC 11322 and *Camp. coli* PCM 2623. Following incubation, diameters of the pathogenic strains’ growth inhibition zones were measured, the slab diameter was subtracted and the results were recorded in mm.

### Determination of *Lactobacillus* Strains Resistance to Bile Salts and Acidic Environment

Resistance to bile salts (Sigma-Aldrich; 1% and 2% *w*/*v* concentration) and low pH level (2 and 3) was analysed based on the method by Zielińska et al. [25] with modifications. A control sample was cultured in 0.85% (w/v) physiological saline solution with a pH of 5.5.

After 24 h of incubation at 37 °C, the *Lactobacillus* strains were centrifuged at 3468 × g RCF for 10 min (Centrifuge MPW-251; MPW). The biomass was suspended in a physiological saline solution with the addition of bile salts or at specified pH levels and incubated at 37 °C without oxygen limitation. The control sample was also inoculated and incubated under the same conditions.

In the beginning, and after 1, 2 and 4 h, serial dilutions were prepared from each bile salt solution, physiological saline solution with defined pH levels and the control sample. The plate count method was used to determine the number of bacteria in MRS agar. The plates were incubated at 37 °C for 48 h without oxygen limitation; after which time the colonies were counted and the results were given in colony-forming units per ml (CFU/ml).

The *Lactobacillus* spp. monocultures in physiological saline solutions with a certain pH level or with the addition of bile salts, as well as control samples, were conducted in three repetitions.

### Resistance to Antibiotics

In order to assess antibiotic resistance properties of the *Lactobacillus* strains the disc diffusion method of Halder et al. was used [26]. Antibiotic susceptibility was tested against compounds categorized as β-lactams (amoxicillin, penicillin G), tetracyclines (doxycycline, tetracycline), macrolides (erythromycin) and aminoglycosides (kanamycin). Oxoid™ paper discs (ThermoFisher Scientific Inc.) were used, with doses of 30 μg of doxycycline, tetracycline, erythromycin and kanamycin, 25 μg of amoxicillin and 10 U of penicillin G.

The spread plate method was used to inoculate the MRS agar with the activated *Lactobacillus* strains. After 30 min the Oxoid™ discs were placed on the surface of the agar in three replications. The plates were incubated for 48 h at 37 °C without oxygen limitation. The susceptibility of the analysed strains was expressed as a diameter of the growth inhibition zone.

### Hydrophobicity

The microbial adhesion to hydrocarbon (MATH) test was used to evaluate the hydrophobic properties of selected *Lactobacillus* strains based on the method first described by Rosenberg et al. [27] with modifications.

Each activated *Lactobacillus* strain’s cells were washed twice with PBS by centrifuging at 3468 × g RCF for 10 min. Afterwards, they were suspended in PBS to yield a final optical density of 1.0– 600 nm, as measured spectrophotometrically (Beckman DU 640, Beckman Coulter Inc., Brea, CA, USA). Each bacterial suspension (5 ml) was mixed with 1 ml of hexadecane (apolar solvent; Sigma-Aldrich) and vortexed for 2 min to the emulsion formation. After 60 min of incubation at room temperature, the PBS fraction absorbance was measured.

Hydrophobic properties of the strains were analysed in three replications, and the cells’ adhesion to the hydrocarbon was calculated by the formula provided by Chae et al. [28]:

$$ Hydrophobicity\ \left(\%\right)=\left[\left({A}_0-\Big(A/{A}_0\right)\right]\times 100. $$

The *Lactobacillus* strains’ affinity to solvent was classified—following Chae et al. [28] with modifications of Ben Taheur et al. [29]—as hydrophilic (< 10%), moderately hydrophilic (10–34%), moderately hydrophobic (35–70%) or highly hydrophobic (71–100%).

### Aggregation and Co-Aggregation Assay with Pathogens

The aggregation assay was performed according to Kos et al. [30]. Biomass of the *Lactobacillus* spp., as well as three *Salmonella* strains and *L. monocytogenes* ATCC 13932, activated monocultures were obtained by centrifuging (3852 × g, 10 min) and then washed once and re-suspended in PBS. A final optical density of cells suspensions was set at 1.0 at 600 nm. Next, the suspensions were vortexed (10 s) and incubated at ambient temperature for 24 h. Afterwards, the absorbance of the upper suspension (100 μl) was measured at 600 nm. The auto-aggregation percentage was determined using the equation:

$$ Auto\hbox{--} aggregation\ \left(\%\right)=\left[1-\left({A}_t/{A}_0\right)\right]\times 100, $$

where A_t_ represents the absorbance after the incubation, whereas A_0_ represents the absorbance at *t* = 0 h.

For the co-aggregation assay, cell suspensions were prepared in the same way as for the auto-aggregation test. Equal volumes of monocultures of *Lactobacillus* spp. and pathogenic bacterial suspensions were mixed by vortexing (10 s), and after 24 h of incubation at ambient temperature, their absorbance was measured (λ = 600 nm). The absorbances of monocultures suspensions from the auto-aggregation assay constituted control samples.

The percentage of co-aggregation was calculated according to Handley et al. [31]:

$$ Co\hbox{--} aggregation\ \left(\%\right)=\frac{\left({A}_x+{A}_y\right)/\left(2-{A}_{\left(x+y\right)}\right)}{\left({A}_x+{A}_y\right)/2}\times 100, $$

where Axe and A_y_ stand for each of the two stsrains in the control samples and A_(x + y)_ represents their mixture. Each experiment was performed in triplicate.

### Adherence to Abiotic and Biotic Surfaces—Biofilm Formation

The ability of the *Lactobacillus* spp. to form biofilm on abiotic (polystyrene and glass) and biotic (collagen, gelatine, and porcine mucous) surfaces was also determined, according to the methods published by Aleksandrzak-Piekarczyk et al. [32] with slight modification.

Suspension of each active *Lactobacillus* strain in PBS were centrifuged at 3468 × g RCF for 10 min and washed, and then the absorbance (A) was adjusted to 1.0 at a wavelength of 630 nm.

The binding assay for polystyrene was performed on unmodified 96-well polystyrene microplates (Corning Inc.), whereas adhesion to glass was analysed with 6-well polystyrene microplates (Corning Inc.) with cover glass placed on the bottom of each well. In order to assess the adhesion ability to collagen, commercially available 96-well polystyrene microplates with a collagen coating were used (Corning® BioCoat™ Collagen I; Corning Inc.). To determine the *Lactobacillus* strains’ adhesion to gelatine and porcine mucous 24-well microplates (Corning Inc.) were coated (60 min, 37 °C and 24 h, 4 °C) with 1% (*w*/*v*) sterile-filtered gelatine (Sigma-Aldrich) or with 150 mg/ml of mucous from porcine stomach solution in PBS (Type II; Sigma-Aldrich; 72 h, 4 °C), respectively. The excess of unbounded gelatine or mucous was removed and wells were washed with PBS.

Bacterial suspensions were added to the microplates in 3 (6-well and 24-well plates) or 8 (96-well plates) repetitions. Different volumes of samples were used depending on the type of plate: 100 μl (96-well microplates), 1 ml (24-well microplates) and 3 ml (6-well microplates). Afterwards, the plates were incubated for 2 h at 37 °C, and unattached cells were washed with PBS. Next, adhered cells were fixed with 80% (*v*/v) methanol (15 min, for polystyrene, glass and collagen adherence assays) or at 60 °C (20 min, for gelatine and porcine mucus adherence assays) and stained with 0.1% (*w*/*v*) crystal violet for 15 min. Subsequently, wells were rinsed with PBS and then the pigment was washed out from attached cells for 45 min on an orbital shaker (Chemland, Stargard Szczeciński, Poland). For pigment removal, 96% (v/v) ethanol (for polystyrene, glass and collagen adherence assays) or 20 mM of citrate buffer (for gelatine and porcine mucus adherence assays) was used.

Absorbance was measured with a TriStar2 S LB 942 microplate reader (Berthold Technologies GmbH & Co. KG) at wavelengths of 490 nm (for polystyrene, glass and collagen adherence assays) or 570 nm (for gelatine and porcine mucus adherence assays). The adhesion ratio was calculated based on the equation:

$$ Adhesion\ ratio={A}_{sample}/{A}_{control}, $$

where A_sample_ is the absorbance of the sample and A_control_ is the control sample’s absorbance (PBS solution added to wells).

### Adherence of Probiotic *Lactobacillu*s Spp. to Caco-2 Cells in the Competition Assay with Pathogens

The adherence assay was performed using Caco-2 cells, which were cultured in 24-well tissue culture plates (Becton, Dickinson and Co., Franklin Lakes, NJ, USA) to obtain confluent monolayers of 2.5 × 10^5^ cells per well. Prior to the experiment, the *Lactobacillus* spp. and the pathogenic bacteria strains—*Salm.* Choleraesuis PCM 2565, *Salm*. Enteritidis ATCC 13076, *Salm*. Typhimurium ATCC 13311 and *L. monocytogenes* ATCC 13932 — were grown for 24 h at 37 °C in MRS broth or nutrient broth, respectively. Next, they were centrifuged (3852×g RCF, 10 min), washed with sterile PBS and re-suspended in fresh DMEM without antibiotics and supplements. Bacterial suspensions were deposited on Caco-2 cell monolayers in the amount of 10^7^ CFU/ml as a single strain (control) or as combinations of monocultures *Lactobacillus* strain and pathogen (1:1, *v*/v) and incubated at 37 °C in an atmosphere of 5% CO_2_ for 2 h. To ensure the same number of bacteria was added to the wells each time, standard curves comparing the amount of each strain and its absorbance (λ = 540 nm) were performed. Each experiment was performed in three replications. After incubation, the unbound microorganisms were aspirated. Caco-2 cells with adhered bacteria were removed from the bottom of wells with 1% (*w*/*v*) trypsin-EDTA (Sigma-Aldrich) for 10 min at 37 °C and sterile cell scrapers (Greiner Bio-One International GmbH, Kremsmünster, Austria), additionally. Subsequently, cells were centrifuged (3852×g RCF, 10 min) and exposed to 0.1% (*v*/v) Triton X-100 (Sigma-Aldrich) for 5 min to lyse Caco-2 cells. The adhered microorganisms were enumerated by the pour plate method on MRS agar (*Lactobacillus* spp.) or nutrient agar (pathogens) media (incubation for 48 h at 37 °C). A ratio of the number of adhered pathogens to the initial number of total pathogenic bacteria added to each well expressed the adherence inhibition rate (%) caused by the *Lactobacillus* spp. presence, which was calculated according to the formula:

$$ Adherence\ inhibition\left(\%\right)=\left[\frac{\log\ \left( adherence\ of\ the\ tested\ sample\right)\times 100}{\log\ \left( adherence\ of\ the\ control\right)}\right]-100. $$

The above procedure for assessing adherence was performed in the Nunc 8-well Lab-Tek TM Chamber Slides system (Thermo Fisher Scientific Inc.). After removing non-adhered microorganisms, wells were washed with PBS and Caco-2 cells were fixed with 80% (*v*/v) methanol (15 min). After air-drying, the 0.1% (*w*/*v*) crystal violet was added to each chamber for 10 min to stain the preparations. Afterwards, wells were washed with 70% (v/v) ethanol until full discolouration and were dried overnight. A phase-contrast microscope (Nikon, Tokyo, Japan) connected to a digital camera (Nikon Digital Sight DS-U3; Nikon) with compatible imaging computer software (NIS-elements BR 3.0; Nikon) was used to observe adherence under 1000× magnification.

### Statistical Analysis

XLSTAT software (Addinsoft, SARL, Paris, France) was used to perform statistical analysis. The data showed herein constitute the arithmetic means of values from three or more repetitions, depending on the assay in question. Before performing analysis of variances (ANOVA; at a significance level of *p* < 0.05), normal distribution (Shapiro-Wilk test) and homogeneity of variances (Bartlett’s test) of the data were confirmed. For antiproliferation activity of metabolites produced by the selected strains towards Caco-2 cells, the results were analysed using one-way ANOVA. The same test was performed to describe the results of the auto-aggregation assay and single probiotic strains’ adhesion to Caco-2 cells. Multi-way ANOVA was carried out for the statistical analysis of data on resistivity to an acidic environment and bile salt concentration, as well as in the case of the co-aggregation assay (two-way ANOVA). Moreover, Tukey’s post hoc test was used after each ANOVA.

## Results

### Safety Assessment

#### Haemolytic Activity

Green halos, with diameters of 21.67 ± 1.53, 21.33 ± 1.53 and 19.67 ± 1.53 mm, were observed when *Lact. pentosus* ŁOCK 1094, *Lact. reuteri* ŁOCK 1092 and *Lact. rhamnosus* ŁOCK 1087, respectively, were grown on the MRS agar with the addition of 5% (*v*/v) defibrinated sheep blood, which indicated α-haemolytic activity. On the other hand, haemolysis was not shown by *Lact. paracasei* ŁOCK 1091 and *Lact. plantarum* ŁOCK 0860.

### Mucin Degradation

Based on the fact that no visible discoloured halos around the colonies were observed, it was found that selected *Lactobacillus* strains cannot degrade mucin.

### Cytotoxicity of BCS towards Caco-2 Cells

**Fig. 1:**
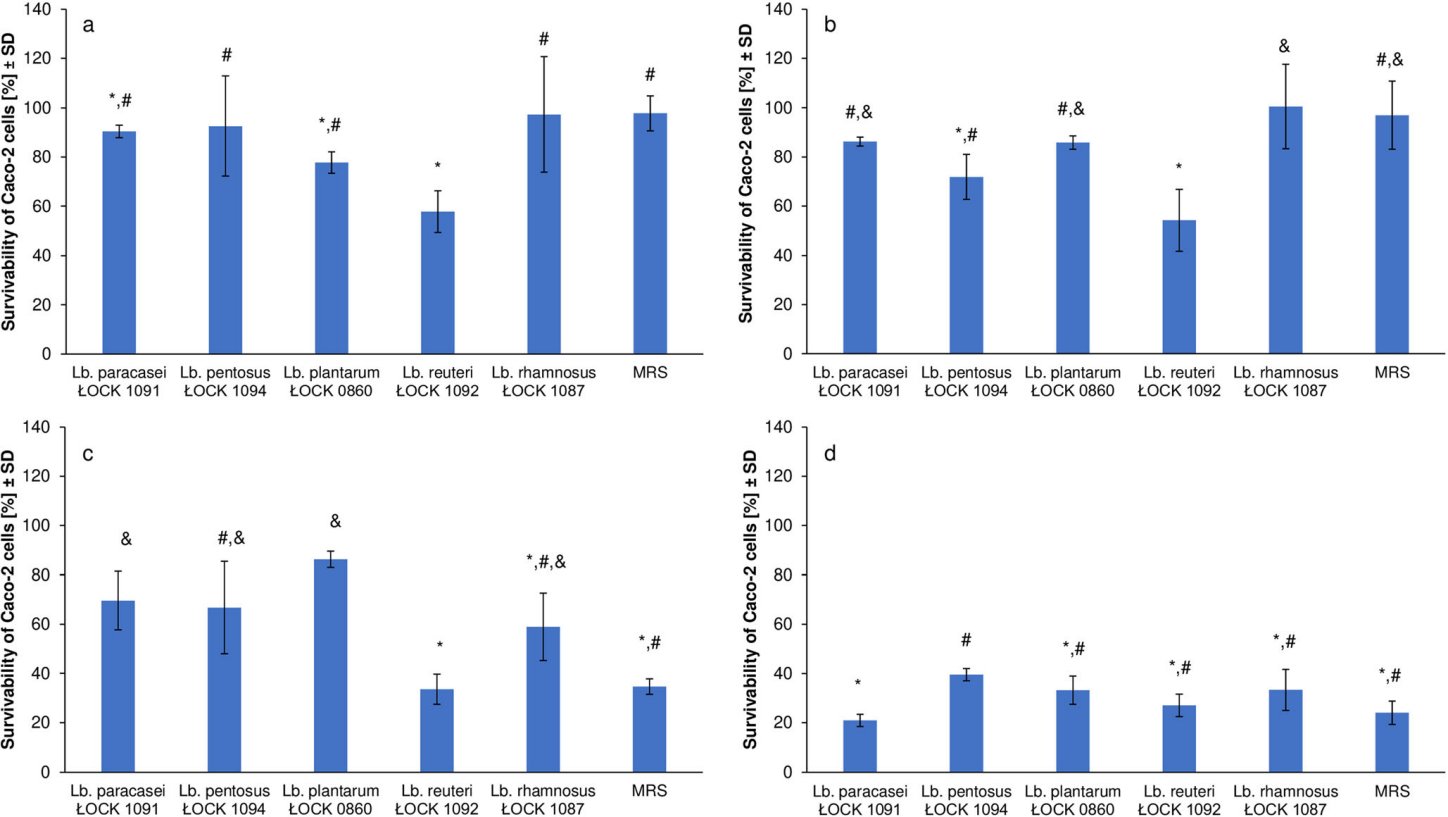
The survivability of Caco-2 cells in the presence of bacterial culture supernatants (BCS) of *Lactobacillus* spp. in different concentration, namely A – 5% (*v*/v), B – 10% (v/v), C – 20% (v/v) and D – 50% (v/v). Moreover, different statistic analysis were used, for each data set, viz. A – Welsh-ANOVA test, B – ANOVA test, C and D – Kruskal-Wallis test, where lowercase letters (a-d) represents significantly different outcomes.

The effect of the *Lactobacillus* strains’ metabolites on the survivability of Caco-2 cells was analysed, the results are presented in Fig. 1.

Metabolites produced by most of the analysed strains, except *Lact. reuteri* ŁOCK 1092, showed weak or no cytotoxic potential towards Caco-2 cells when the BCS was used in concentrations of 5% and 10% (*v*/v), the effect was comparable to that of the control (un-inoculated MRS). The survivability of human colon adenocarcinoma cells exceeded 54% (Figs. 1a and 1b).

The survivability of Caco-2 cells varied from 58.95 ± 13.70% to 86.22 ± 3.31% when the 20% (v/v) BCS of most of the selected strains was incorporated. This was significantly higher than that of the control (34.64 ± 3.16%). The cytotoxic effect of *Lact. reuteri* ŁOCK 1092 BCS at a concentration of 20% (v/v) was similar to the one observed for pure MRS (Fig. 1c).

The effect of higher BCS concentration (50% v/v) of all strains on Caco-2 cells’ survivability was comparable to that observed for un-inoculated MRS (Fig. 1d). Hence, in comparison to the cytotoxicity of pure (un-inoculated) MRS medium, the tested BCS demonstrated no or weak cytotoxic action towards Caco-2 cells.

### Probiotic Properties Assessment

#### Antagonistic Properties of Probiotic *Lactobacillus* Spp. Against Pathogenic Bacteria

**Fig. 2:**
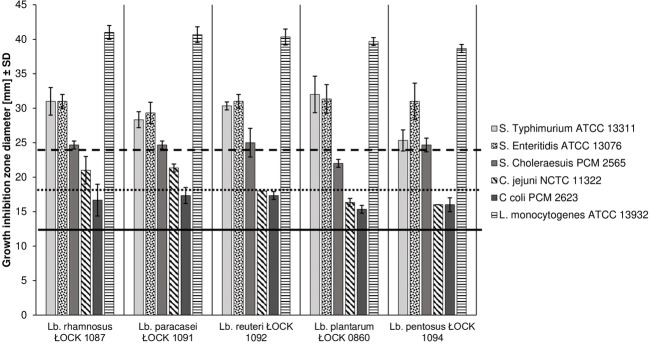
Antagonistic activity of *Lactobacillus* spp. strains against pathogenic bacteria. Inhibition type: above the dashed line (≥23 mm) - strong; between dotted (≥17 mm) and dashed (<23 mm) lines - moderate; between continuous (≥11 mm) and dotted (<17 mm) line - weak; below continuous black line (<11 mm) - no inhibition. Data from three replicates (± SD). The strength of inhibition was evaluated according to Tsai et al. (2005) [33].

All *Lactobacillus* strains, in the study, demonstrated strong antagonistic activity towards the *Salmonella* strains and *L. monocytogenes* ATCC 13932. The exception was *Lact. plantarum* ŁOCK 0860 which showed only moderate inhibition of *Salm.* Choleraesuis PCM 2565. *Camp. jejuni* NCTC 11322 growth was weakly inhibited by *Lact. plantarum* ŁOCK 0860 and *Lact. pentosus* ŁOCK 1094 which, along with *Lact. rhamnosus* ŁOCK 1087, also had a slight effect on *Camp. coli* PCM 2623 growth. However, moderate antagonistic properties of *Lact. rhamnosus* ŁOCK 1087 were observed against *Camp. jejuni* PCM 2623. Both of analysed *Campylobacter* strains growth was moderately influenced by *Lact. paracasei* ŁOCK 1091 and *Lact. reuteri* ŁOCK 1092. The results are presented in Fig. 2.

### The Resistance of the *Lactobacillus* Spp. to Bile Salts and Acidic Environment

Based on the results obtained, it was noted that the resistivity of analysed probiotic strains to an acidic environment was high, the survival of the bacteria exceeded 88% up to 4 h of incubation (Table 1). The resistance of the strains to an acidic environment was strain-specific, and significantly higher resistivity was observed for *Lact. plantarum* ŁOCK 0860 and *Lact. rhamnosus* ŁOCK 1087. In addition, selected probiotic strains demonstrated greater capability of surviving in pH 3.0 rather than pH 2.0. Moreover, the strains were able to survive in the presence of 1% (*w*/*v*) and 2% (w/v) bile salts in which the bacterial population density was reduced by no more than 10% after 4 h of incubation (Table 2).

**Table 1:** Probiotic *Lactobacillus* strains survivability in an acidic environment

*Lactobacillus* strain	pH							
	2				3			
	Time [h]] ± SD *							
	0	1	2	4	0	1	2	4
	Cell survival rate [%]							
*paracasei* ŁOCK 1091	100 ^d^	94.39 ± 1.26 ^a, b, c, d^	93.82 ± 1.76 ^a, b, c, d^	91.39 ± 3.16 ^a, b, c, d^	100 ^d^	95.72 ± 1.26 ^a, b, c, d^	94.92 ± 1.21 ^a, b, c, d^	93.44 ± 0.45 ^a, b, c, d^
*pentosus* ŁOCK 1094		94.91 ± 2.41 ^a, b, c, d^	92.92 ± 3.58 ^a, b, c, d^	91.17 ± 3.98 ^a, b, c^		96.79 ± 2.51 ^a, b, c, d^	95.28 ± 1.14 ^a, b, c, d^	94.19 ± 1.83 ^a, b, c, d^
*plantarum* ŁOCK 0860		97.25 ± 2.75 ^b, c, d^	92.88 ± 1.96 ^a, b, c, d^	91.08 ± 0.73 ^a, b, c^		98.32 ± 2.50 ^b, c, d^	95.97 ± 1.66 ^a, b, c, d^	94.14 ± 3.01 ^a, b, c, d^
*reuteri* ŁOCK 1092		92.74 ± 3.26 ^a, b, c, d^	90.15 ± 1.62 ^a, b^	88.12 ± 2.37 ^a^		94.68 ± 2.35 ^a, b, c, d^	91.65 ± 0.69 ^a, b, c, d^	89.70 ± 1.31 ^a, b^
*rhamnosus* ŁOCK 1087		96.18 ± 1.42 ^a, b, c, d^	95.02 ± 2.41 ^a, b, c, d^	90.96 ± 1.19 ^a, b, c^		98.98 ± 2.83 ^c, d^	97.07 ± 2.32 ^b, c, d^	95.76 ± 1.77 ^a, b, c, d^

*Mean values labelled by various lowercase letters (a, b, c, d) were significantly different (multi-way ANOVA with post hoc Tukey’s test; p < 0.05). Multi-way ANOVA included the influence of used strains, pH levels and time of incubation, as well as interactions of these explanatory variables on the survival of analysed probiotics in the acidic environment

**Table 2:** Probiotic *Lactobacillus* strains survival in the presence of bile salts

*Lactobacillus *strain	Bile salt concentration [% w/v]							
	1				2			
	Time [h]							
	0	1	2	4	0	1	2	4
	Cell survival rate [%] ± SD *							
*paracasei* ŁOCK 1091	100 ^b^	96.96 ± 2.63 ^a, b^	95.73 ± 3.22 ^a, b^	95.33 ± 1.19 ^a, b^	100 ^b^	95.86 ± 2.21 ^a, b^	94.61 ± 2.52 ^a, b^	93.68 ± 1.31 ^a, b^
*pentosus* ŁOCK 1094		98.09 ± 2.32 ^a, b^	95.95 ± 1.03 ^a, b^	92.87 ± 1.78 ^a, b^		95.85 ± 3.39 ^a, b^	93.84 ± 2.74 ^a, b^	90.93 ± 1.17 ^a^
*plantarum* ŁOCK 0860		99.67 ± 1.37 ^a, b^	98.60 ± 1.91 ^a, b^	96.64 ± 4.45 ^a, b^		98.99 ± 3.28 ^a, b^	97.83 ± 3.52 ^a, b^	94.74 ± 3.62 ^a, b^
*reuteri* ŁOCK 1092		99.14 ± 2.98 ^a, b^	98.71 ± 3.62 ^a, b^	97.70 ± 2.14 ^a, b^		98.13 ± 3.31 ^a, b^	96.85 ± 2.09 ^a, b^	92.37 ± 1.62 ^a, b^
*rhamnosus* ŁOCK 1087		98.35 ± 4.46 ^a, b^	96.65 ± 1.86 ^a, b^	95.97 ± 2.84 ^a, b^		97.16 ± 1.00 ^a, b^	95.27 ± 2.23 ^a, b^	93.28 ± 2.85 ^a, b^

*Mean values labelled by various lowercase letters (a, b) were significantly different (multi-way ANOVA with post hoc Tukey’s test; p < 0.05). Multi-way ANOVA included the influence of used strain, bile salts concentrations and time of incubation, as well as interactions of these explanatory variables on the survival of analysed probiotics in the acidic environment

### Antibiotic Susceptibility of the *Lactobacillus* Strains

**Fig. 3:**
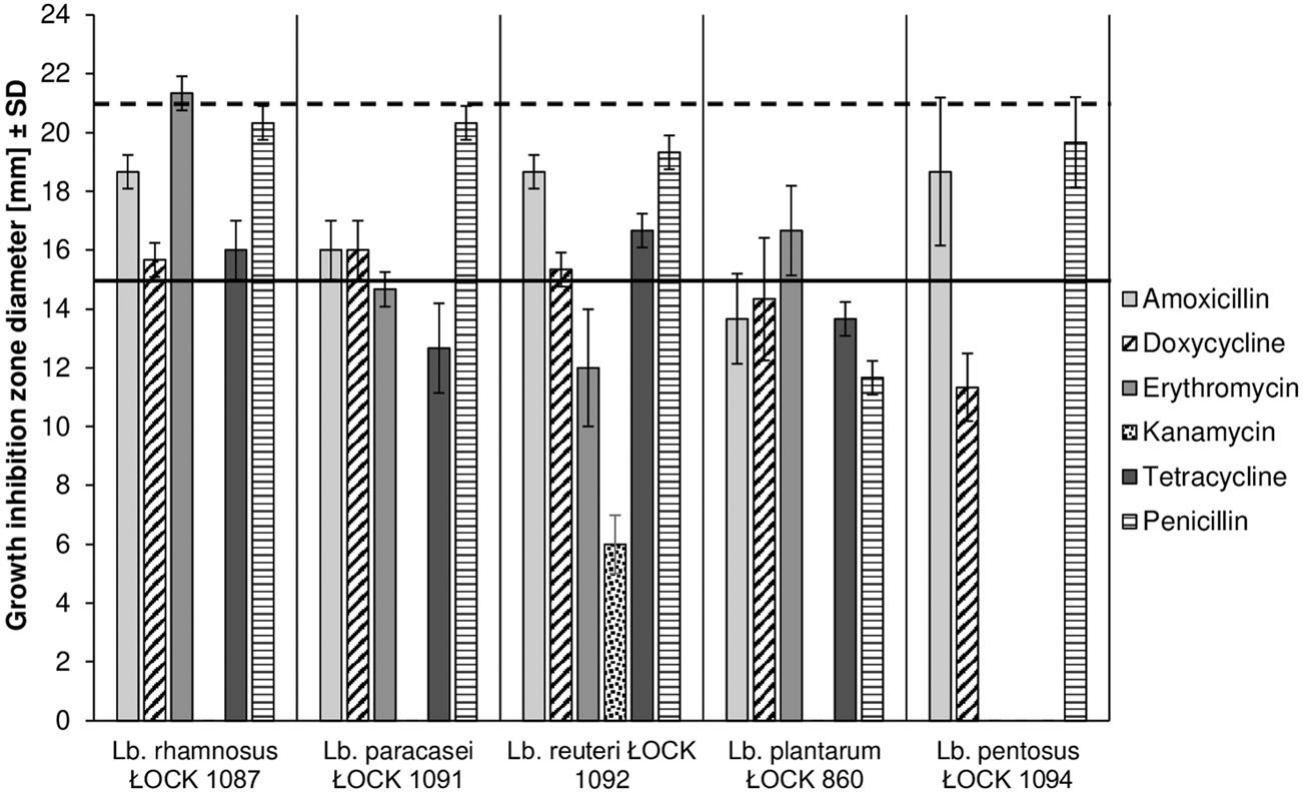
Susceptibility of selected *Lactobacillus* strains to antibiotics. Diameter of growth inhibition zone <15 mm (below continuous black line) indicated to antibiotic resistance; if ranged between continuous line (≥ 15 mm) and dashed line (< 21 mm) – moderate susceptibility; growth inhibition zone diameter ≥ 21 mm (above dashed line) suggested strain being prone to antibiotic [26]. Results are the mean diameter of growth inhibition zone from three repetitions (± SD) with antibiotic disc diameter being included.

Selected *Lactobacillus* strains displayed various levels of antibiotic resistance, ranging from strong resistivity to moderate susceptibility (Fig. 3). All of the *Lactobacillus* strains showed resistivity against kanamycin. Each of the probiotic strains was moderately susceptible to amoxicillin and penicillin, which caused a growth inhibition zone ranging between 13 and 19 mm (mean: 17 mm) and 12–20 mm (mean: 18 mm), respectively. *Lact. rhamnosus* ŁOCK 1087 was highly sensitive to erythromycin, which did not affect *Lact. paracasei* ŁOCK 1091, *Lact. reuteri* ŁOCK 1092 and *Lact. pentosus* ŁOCK 1094. A moderate inhibition zone, with a diameter of 17 mm, was observed when erythromycin interacted with *Lact. plantarum* ŁOCK 0860. Furthermore, *Lact. rhamnosus* ŁOCK 1087, *Lact. paracasei*, ŁOCK 1091 and *Lact. reuteri* ŁOCK 1092 were moderately susceptible to doxycycline and tetracycline, except *Lact. paracasei* ŁOCK 1091 strain, which was resistant to tetracycline. Neither of these antibiotics had an impact on the growth of *Lact. plantarum* ŁOCK 0860 or *Lact. pentosus* ŁOCK 1094.

### Hydrophobicity

The hydrophobicity of selected *Lactobacillus* strains was varied, ranging from 12.80% ± 1.77% to 41.14% ± 0.83% (mean: 24.39%). *Lact. rhamnosus* ŁOCK 1087, *Lact. paracasei* ŁOCK 1091, *Lact. plantarum* ŁOCK 0860 and *Lact. pentosus* ŁOCK 1094 were classified as moderately hydrophilic with the adherence to hexadecane of 25.12% ± 1.33%, 12.80% ± 1.77%, 27.17% ± 0.84% and 33.50% ± 1.49%, respectively. Only *Lact. reuteri* ŁOCK 1092 exhibited moderate hydrophobicity towards hexadecane (41.14%).

### Auto- and Co-Aggregation Assessment

Analysed *Lactobacillus* strains, as well as the pathogenic ones, demonstrated the ability to auto-aggregate after 24 h of incubation with an auto-aggregation rate over 50% (Table 3). Among all of the selected strains, the strongest auto-aggregation was observed for *Lact. plantarum* ŁOCK 0860 (95.46% ± 1.51%). Auto-aggregation of the tested pathogens was high, reaching an average of 71.61% and significantly stronger than capability observed for *Lact. paracasei* ŁOCK 1091 (56.56% ± 4.71%), *Lact. reuteri* ŁOCK 1092 (54.65% ± 2.97%) and *Lact. rhamnosus* ŁOCK 1087 (54.85% ± 2.44%). The exception was *Salm*. Choleraesuis PCM 2565 which auto-aggregation ability was comparable (60.63% ± 0.93%) to the one exhibited by the above-mentioned strains.

**Table 3:** Auto-aggregation ability of *Lactobacillus* spp., *L. monocytogenes* and *Salmonella* spp. Data from three experiments (± SD)

Probiotic strains	Strain	Auto-aggregation [%] ± SD *
	*Lact. paracasei* ŁOCK 1091	56.56 ± 4.71 ^a^
	*Lact. pentosus* ŁOCK 1094	73.97 ± 3.75 ^b^
	*Lact. plantarum* ŁOCK 0860	95.46 ± 1.51 ^c^
	*Lact. reuteri* ŁOCK 1092	54.65 ± 2.97 ^a^
	*Lact. rhamnosus* ŁOCK 1087	54.85 ± 2.44 ^a^
Pathogens	*L. monocytogenes* ATCC 13932	70.02 ± 1.57 ^b^
	*Salm*. Choleraesuis PCM 2565	60.63 ± 0.93 ^a^
	*Salm.* Enteritidis ATCC 13076	73.68 ± 2.94 ^b^
	*Salm*. Typhimurium ATCC 13311	74.82 ± 1.09 ^b^

*Mean values marked with different lowercase letters (a, b, c) were significantly different (one-way ANOVA with post hoc Tukey’s test; p < 0.05)

Furthermore, the *Lactobacillus* strains’ co-aggregation with pathogenic bacteria was evaluated (Table 4). Most of the probiotic strains exhibited the highest degree of co-aggregation with *Salm.* Choleraesuis PCM 2565 ranging between 66.64% ± 4.08% and 87.75% ± 0.88% (mean: 76.84%). However, *Lact. rhamnosus* ŁOCK 1087 showed the highest co-aggregation with *L. monocytogenes* ATCC 13932 (70.90% ± 0.91%). The co-aggregation ability of the *Lactobacillus* spp. with *Salm*. Typhimurium ATCC 13311 and *Salm*. Enteritidis ATCC 13076 was significantly weaker, varying from 12.12% ± 2.55% to 65.24% ± 3.28% (mean: 40.10%) and from 34.16% ± 1.52 to 66.60% ± 4.72% (mean: 46.87%), respectively.

**Table 4:** Co-aggregation of probiotic *Lactobacillus* strains with pathogenic bacteria from *Salmonella* genus and *L. monocytogenes*

Pathogen	Probiotic strain				
	*Lact. paracasei* ŁOCK 1091	*Lact. pentosus* ŁOCK 1094	*Lact. plantarum* ŁOCK 0860	*Lact. reuteri* ŁOCK 1092	*Lact. rhamnosus* ŁOCK 1087
	Co-aggregation [%] ± SD *				
*L. monocytogenes* ATCC 13932	58.97 ± 6.00 e, f	82.19 ± 3.62 i	67.55 ± 0.61 f, g	70.31 ± 2.93 g, h	70.90 ± 0.91 g, h
*Salm*. Choleraesuis PCM 2565	67.09 ± 7.06 f, g	85.45 ± 1.23 i	79.02 ± 2.14 h, i	75.12 ± 2.65 g, h, i	66.64 ± 4.08 f, g
*Salm.* Enteritidis ATCC 13076	40.17 ± 3.38 b, c, d	49.94 ± 0.60 e	34.16 ± 1.52 b, c	66.60 ± 4.72 f, g	43.50 ± 4.50 c, d
*Salm*. Typhimurium ATCC 13311	65.24 ± 3.28 f, g	30.80 ± 4.70 b	12.12 ± 2.55 a	47.37 ± 3.38 d	44.99 ± 3.64 c, d

*Significantly different results were labelled with a various lowercase letter (a – i) according to data obtained from two-way ANOVA (p < 0.05) with the post hoc Tukey’s test. Two-way ANOVA considered the interaction between categorical variables, namely, probiotic and pathogenic strain

### Biofilm Formation by *Lactobacillus* Spp.

Based on the results, it can be concluded that selected *Lactobacillus* strains showed weak or moderate adherence to the tested surfaces (Table 5). The probiotic strains’ adhesion to polystyrene and collagen was weak except for *Lact. rhamnosus* ŁOCK 1087 which did not form a biofilm on the polystyrene surface. The binding of the probiotic cells to glass and porcine mucus ranged from weak to moderate adhesion strength. Nevertheless, all of the analysed strains exhibited strong adhesion to gelatine.

**Table 5:** Biofilm formation properties of probiotic *Lactobacillus* strains

Lactobacillus strain	Adhesion ± SD to different surfaces *				
	polystyrene	glass	gelatine	collagen	porcine mucus
*paracasei* ŁOCK 1091	1.01 ± 0.15 +	2.44 ± 0.14 ++	7.16 ± 0.16 +++	1.16 ± 0.14 +	1.87 ± 0.07 +
*pentosus* ŁOCK 1094	1.33 ± 0.12 +	1.18 ± 0.27 +	2.92 ± 0.06 +++	1.23 ± 0.15 +	2.06 ± 0.10 ++
*plantarum* ŁOCK 0860	1.21 ± 0.10 +	2.15 ± 0.12 ++	8.31 ± 0.28 +++	1.34 ± 0.13 +	2.70 ± 0.09 ++
*reuteri* ŁOCK 1092	1.14 ± 0.14 +	2.32 ± 0.16 ++	5.50 ± 0.13 +++	1.20 ± 0.14 +	2.02 ± 0.08 ++
*rhamnosus* ŁOCK 1087	0.95 ± 0.08 -	1.95 ± 0.10 +	6.03 ± 0.24 +++	1.04 ± 0.15 +	2.10 ± 0.12 ++

*Ability of *Lactobacillus* spp. to adhere to different surfaces and thus forming biofilm was evaluated based on do Carmo et al. (2016). Adherence of the tested sample (A_sample)_ was compared with adherence of the control (A_control_) and strains were classified as follows: “-“non-adherent – A_sample_ < A_control_; “+” weakly adherent – A_control_ < A_sample_ ≤ (2 × A_control_); “++” moderately adherent – (2 × A_control_) < A_sample_ ≤ (4 × A_control_); “+++” strongly adherent – (4 × A_control_) < A_sample_

### Adherence of Probiotic *Lactobacillus* Strains to Caco-2 Cells

In this assay, bacteria were incubated with Caco-2 cells monolayers for 2 h—during that time no morphological changes (no cytotoxicity) were observed (with the inverted microscope), either in the cells or in the monolayer.

**Fig. 4:**
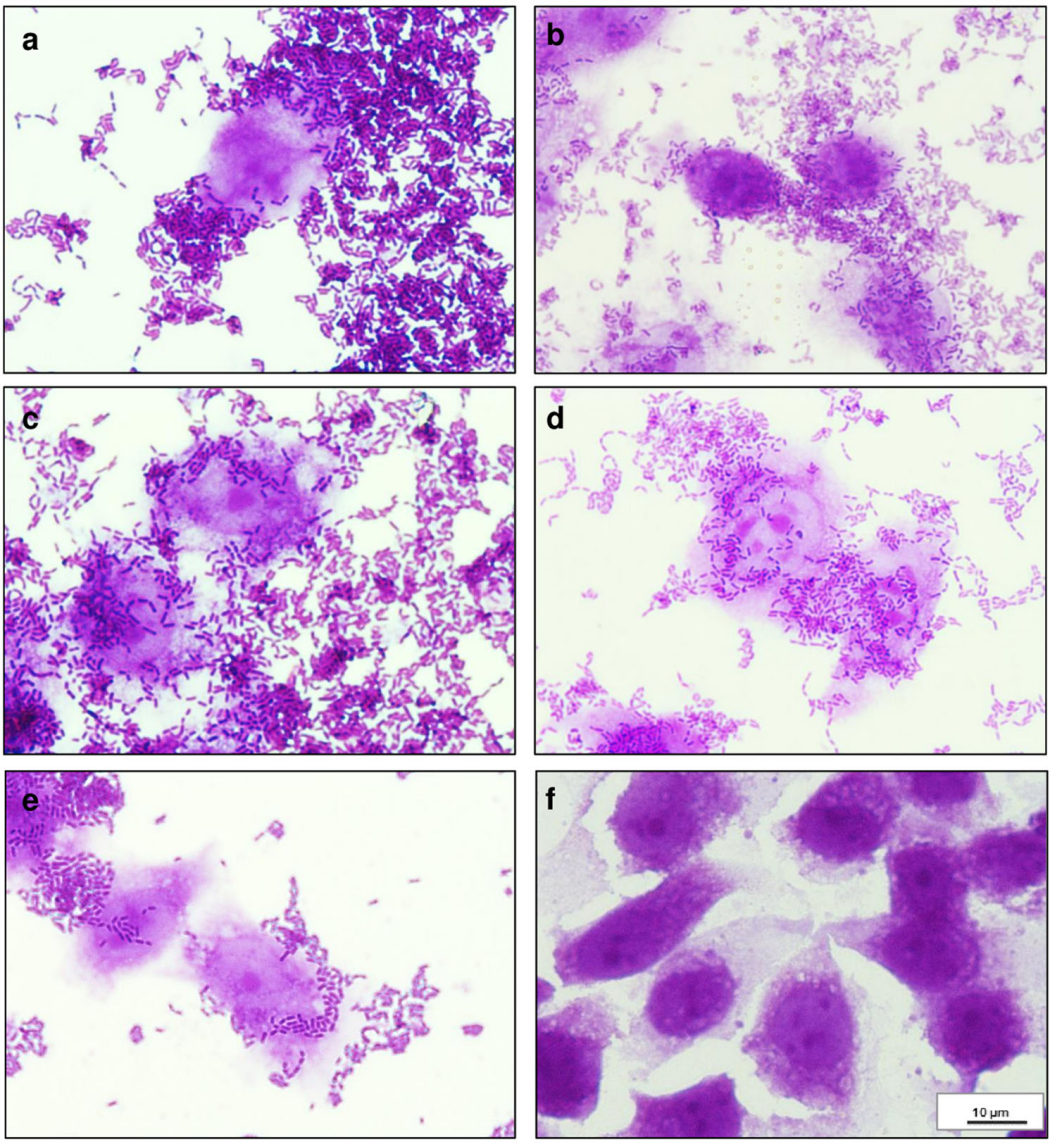
Adherence of (A) *Lact. plantarum* ŁOCK 0860, (B) *Lact. paracasei* ŁOCK 1091, (C) *Lact. reuteri* ŁOCK 1092, (D) *Lact. pentosus* ŁOCK 1094, (E) *Lact. rhamnosus* ŁOCK 1087 to human colon adenocarcinoma cell line Caco-2 (F: control), after a 2 h incubation (1000 ×, Nikon – Eclipse, Japan) stained with crystal violet.

The number of adhered bacteria per Caco-2 cell is the basis of the adhesion type classification described by Saidi et al. [34] according to which *Lact. pentosus* ŁOCK 1094 and *Lact. plantarum* ŁOCK 0860 were classified as moderately adhesive (Table 6). On the other hand, on average 16.35 and 14.97 cells of *Lact. rhamnosus* ŁOCK 1087 and *Lact. reuteri* ŁOCK 1092, respectively, adhered to one Caco-2 cell, which was classified as weak adhesion. *Lact. paracasei* ŁOCK 1091 were classified as non-adhesive. Nevertheless, the adhesion rates of the *Lactobacillus* spp. were high, exceeding 85%. The results of the microscopic observations of adhesion are presented in Fig. 4.

**Table 6:** Adherence of *Lactobacillus* spp. to the human colon adenocarcinoma cell line Caco-2. Data from three independent experiments (± SD)

Strain	Adherence rate [%] ± SD	Number of adhered bacterial cells / 1 Caco-2 cell	Adhesion type classification *
*Lact. paracasei* ŁOCK 1091	85.09 ± 4.08	6.53	NA
*Lact. pentosus* ŁOCK 1094	95.05 ± 3.66	34.80	M
*Lact. plantarum* ŁOCK 0860	92.40 ± 2.58	22.28	M
*Lact. reuteri* ŁOCK 1092	90.03 ± 1.36	14.97	W
*Lact. rhamnosus* ŁOCK 1087	90.55 ± 0.72	16.35	W

*Lactobacillus strains were classified as non-adhesive (NA: 0–10), weakly adhesive (W: 10–20), moderately adhesive (M: 20–50) and strongly adhesive (S: > 50), according to Saidi et al. [34]

### Competition Assay

**Fig. 5:**
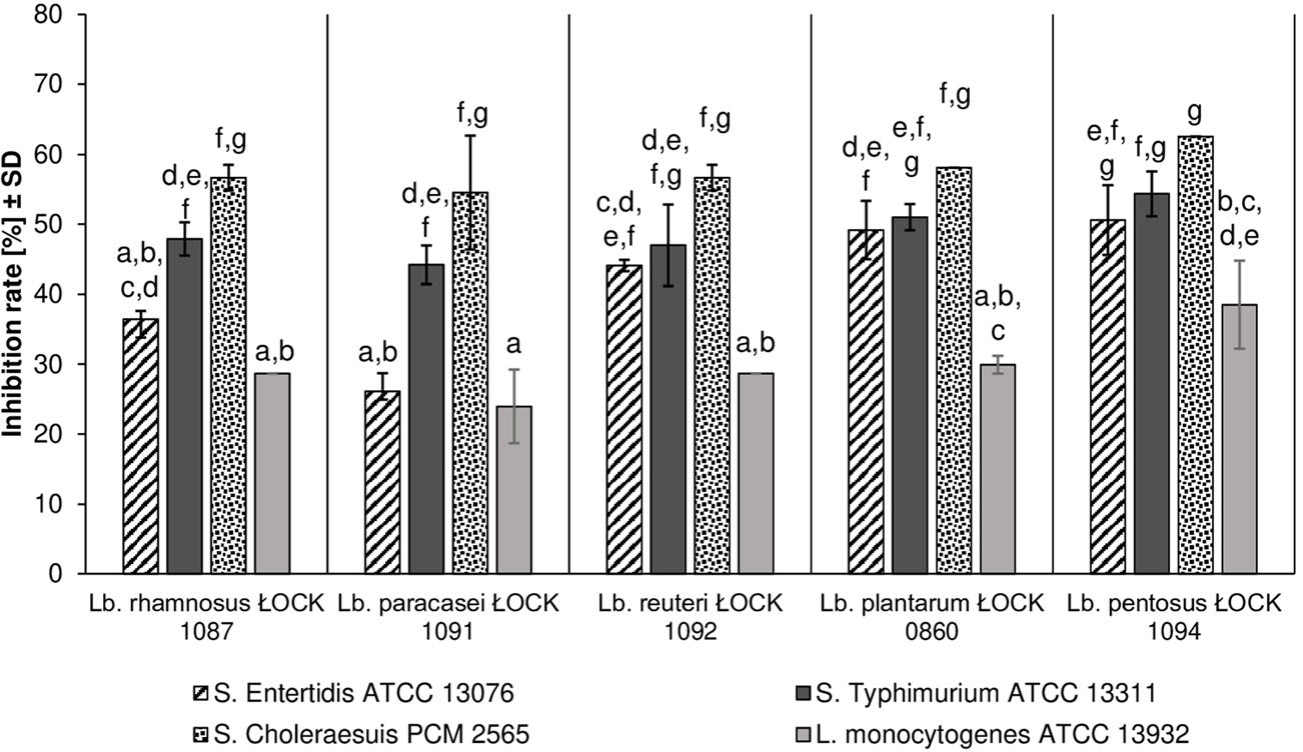
The effect of *Lactobacillus* strains on the adhesion of pathogenic bacteria to human colon adenocarcinoma cell line – Caco-2 expressed as the inhibition rate (%). Displayed data constitute the mean value from three repetitions (± SD). Mean values, which were significantly different were marked with various lowercase letters (a – g), according to the performed two-way ANOVA (*p* < 0.05) results with the post hoc Tukey’s test (p < 0.05). Both explanatory variables, namely probiotic and pathogenic strain bring significant information, as well as the interaction of these categorical variables (probiotic strain*pathogenic strain), therefore results should not be considered separately. Nonetheless, from all explanatory variables, the pathogenic strain was the most influential in competition assay results.

In the competition test with pathogens, all of the tested probiotic strains reduced the adhesion of all pathogenic bacteria. The attachment of *Salm*. Choleraesuis PCM 2565 was inhibited the most significantly (up to 62.58%), while *L. monocytogenes* ATCC 13932 adhesion was prevented the least (from 23.94 ± 5.27% to 38.48 ± 6.28%). The adherence of pathogenic strains was hindered to the greatest extent by *Lact. pentosus* ŁOCK 1094, while *Lact. paracasei* ŁOCK 1091 caused the poorest reduction of pathogen attachment (Fig. 5).

## Discussion

The overuse of antibiotics in livestock breeding has led to the emergence of antimicrobial resistance as a consequence of which the legislative prohibition of AGPs was introduced in the European Union in 2006 [35]. Removing AGPs from animals’ diets has put them at a higher risk of being infected by pathogens; therefore, alternatives to control their prevalence in livestock are being sought [18, 36]. In this paper, the probiotic properties of selected *Lactobacillus* strains intended to be used in monogastric animals (swine and poultry) are described.

Even though the bacteria belonging to *Lactobacillus* species are ‘generally recognized as safe’ (GRAS), in recent years, in vitro assessment of newly introduced probiotics has become necessary [37, 38]. Haemolytic activity and mucous degradation should be evaluated as safety aspects of probiotic candidates [18]. As opposed to α-haemolysis, β-haemolytic activity is considered harmful. Strains exhibiting this capability are known for the production of exotoxin, which lyses blood cells and therefore impacts the immune system [39]. Tested *Lactobacillus* spp. did not show β-haemolytic activity or mucinolytic activity, a finding which is in line with the data described by Adimpong et al. [39] and Ficoseco et al. [40] and that of Nallala et al. [41] and Abouloifa et al. [42], respectively. Moreover, the survivability of Caco-2 cells in the presence of metabolites produced by selected *Lactobacillus* strains was analysed using the NRU test. Our results indicated that only metabolites (BCS) of *Lact. reuteri* ŁOCK 1092 in concentrations of 5% (*v*/v), 10% (v/v) and 20% (v/v) showed a significantly higher anti-proliferative effect towards Caco-2 cells, compared with the control. Bhat et al. [38] reported that Caco-2 cells were not affected by *Lact. rhamnosus* MTCC-5897, a finding which the researchers perceived as the evidence of the strain’s safety and which is in line with our results if compared with pure MRS. Therefore, it can be concluded that the *Lactobacillus* strains can be considered safe since they exhibited no or only weak cytotoxic potential towards human intestinal Caco-2 cells when compared with a pure MRS medium.

Probiotic microorganisms have to be able to survive the internal environment of the host, including gastric acids and bile salts, in order to reach and colonize the small intestine and the colon, as well as to be metabolically active [43]. The selected strains demonstrated high survivability in the presence of bile salts and an acidic environment (above 88%). The probiotic strains in the current study were able to survive in higher levels of bile salts than lactobacilli strains analysed by Owusu-Kwarteng et al. [44] or Gharbi et al. [45].

Microbial isolates intended to be used as probiotics should also display some antibiotic resistance, to survive in the GIT of the host and to exert beneficial activities on it [46]. Five selected *Lactobacillus* strains showed high resistance to kanamycin, which was in line with the results obtained by Mejri and Hassouna [47] and by Casarotti et al. [48]. In addition to that, Hyacinta et al. [49] and Casarotti et al. [48] observed that *Lactobacillus* isolates were sensitive to erythromycin and tetracycline, while this was noted for *Lact. rhamnosus* ŁOCK 1087 in our studies. Nevertheless, *Lact. reuteri* ŁOCK 1092 was also moderately susceptible to tetracycline and *Lact. plantarum* ŁOCK 0860—to erythromycin. Furthermore, it was established that most of the strains under study are moderately sensitive to β-lactams, amoxicillin and penicillin, which was similar to the data presented by Hyacinta et al. [49] and Rao et al. [50]. Moreover, *Lact. plantarum* ŁOCK 0860 and *Lact. pentosus* ŁOCK 1094 were resistant to doxycycline, whereas the rest of the strains were moderately susceptible; these results differed from those obtained by Sharma et al. [51]. Based on the results of the present study, it can be inferred that selected probiotic strains are more susceptible to antibiotics that inhibit bacterial cell wall synthesis, such as β-lactams (amoxicillin and penicillin G), rather than macrolides (erythromycin), tetracyclines (tetracycline and doxycycline) or aminoglycosides (kanamycin), which interfere with protein biosynthesis in cells [52, 53].

Moreover, the strains were screened for their capability to suppress the growth of pathogenic bacteria, one of the crucial factors which determine whether the candidate microbes could be considered for use as probiotics [54]. Dec et al. [55] observed that *Camp. jejuni* strains were more susceptible to *Lactobacillus* isolates than *Camp. coli* in an agar slab assay; however, the sensitivity of the pathogens was mostly moderate, which was similar to our results. Furthermore, Klose et al. [56], who used the agar spot test, noted varied growth inhibition of *Salm*. Choleraesuis DSM 554, *Salm*. Enteritidis USDA 59 and *Salm*. Typhimurium USDA 554 caused by *Lactobacillus* isolates of animal origin. Most of the strains, analysed in that study, exhibited the strongest antagonism towards *Salm*. Choleraesuis DSM 554, whereas in our study, the selected strains showed the strongest inhibition of *Salm*. Typhimurium ATCC 13311 and *Salm*. Enteritidis ATCC 13076 growth [56]. Nonetheless, among the pathogens screened, the highest antagonistic activity by *Lactobacillus* spp. was displayed against *L. monocytogenes* ATCC 13932, which is comparable to the results obtained by Shokryazdan et al. [57] despite different methods used to assess antimicrobial activity.

Antagonistic properties of probiotic strains are also related to auto- and co-aggregation capabilities, as well as to the ability to attach to the intestine with the potential of adhesion inhibition of pathogenic microorganisms [58]. The ability to auto-aggregate is a vital feature, in that it allows probiotics to maintain a significant number of cells in the environmental niche, such as GIT mucose, and to form a barrier against pathogenic microbes [59, 60]. In comparison to the results obtained by Ait Seddik et al. [61], as well as Saini and Tomar [62], the probiotic strains used in our studies exhibited higher auto-aggregation capabilities, even up to 95%. Furthermore, the formation of co-aggregates with pathogens hinders their adhesion to the intestinal epithelium and forms a microenvironment, which helps probiotics to eliminate pathogenic microorganisms [63]. The probiotics aggregated with *L. monocytogenes* ATCC 13932 at the level of 69.98% on average, which was in line with the data described by de Souza et al. [64]. However, strains analysed by Kaktcham et al. [65] exhibited a significantly lower predisposition to co-aggregate with *L. monocytogenes* CFQ-103 (up to 19.15%), which indicates the strain-specific character of this feature. Co-aggregation of the selected probiotics with *Salm*. Enteritidis ATCC 13076 (up to 66.60%) was substantially lower than in case of *L. monocytogenes* ATCC 13932, similar to the results of Tareb et al. [66] but contrary to the results presented by Campana et al. [67]. Moreover, the *Lactobacillus* strains analysed by the above-mentioned research teams showed less ability to aggregate with *Salm*. Enteritidis ATCC 13076, not surpassing 26% [66, 67]. Additionally, Gómez et al. [68] performed a co-aggregation assay for *Lactobacillus* isolates with *Salm*. Typhimurium ATCC 14028 and *L. monocytogenes* ATCC 7644. These researchers’ results indicated the higher aggregation efficiency of lactobacilli with *L. monocytogenes* ATCC 764 than with *Salm*. Typhimurium ATCC 14028, which was comparable to our results [68]. Nevertheless, in contrast to the data described by Sirichokchatchawan et al. [7], we noted that selected probiotic strains exhibited the highest co-aggregation ability with one of the tested *Salmonella* strains, namely, *Salm*. Choleraesuis PCM 2565 (on average, 74.66%), which confirms that the aggregation properties are strain-related.

The adhesion of bacterial cells to the intestinal mucose is of vital importance, not only because of intestinal colonization and the exclusion of pathogens but also in terms of immunomodulation and the synthesis of beneficial bacterial molecules [61]. The adherence rate of the *Lactobacillus* strains in our study exceeded 85% and was substantially higher than that observed for the isolates studied by Feng et al. [69]. However, the rates were comparable to the described by Archer et al. [70], which suggested that the adherence ability of *Lactobacillus* spp. bacteria is strain-specific. Furthermore, selected probiotic strains not only exhibited a predisposition to adhere to epithelial cells but also to hinder the attachment of pathogenic bacteria to Caco-2 cells by up to approximately 60%. Campana et al. [67], as well as Jessie Lau and Chye [71], observed the capability of different *Lactobacillus* isolates to inhibit the adherence of *Salm*. Enteritidis ATCC 13076 and *L. monocytogenes* ATCC 7644 or ATCC 13932, respectively, to varying degrees. These results were partially in line with ours; although, the strains used in our studies showed significantly higher inhibition properties towards *Salm*. Enteritidis ATCC 13076 than *L. monocytogenes* ATCC 13932, which was not noted by the above-mentioned researchers. Albeit, the strongest competition for adherence to Caco-2 cells was observed between the probiotic strains and *Salm*. Choleraesuis PCM 2565, as well as *Salm*. Typhimurium ATCC 13311. The Caco-2 cell-binding assay suggested that the *Lactobacillus* strains possess the potential to compete with pathogens in the GIT, thus facilitating their excretion.

The first contact between microorganisms and the host’s cell walls might be facilitated by features of the microbes’ cell surfaces, such as hydrophobicity; however, this does not ensure strong adhesion. Nevertheless, this characteristic can be influential on aggregation and adhesion capabilities [64]. Although selected probiotic strains were mostly hydrophilic, with a hydrophobicity below 40%, we observed strong auto- and co-aggregation capacities, as well as the ability to attach to Caco-2 cells strongly enough to inhibit pathogens’ adhesion, which was in line with the data described by Ramos et al. [72] and Kim and Baik [73].

Additionally, biofilm formation on biotic (gelatine, collagen and porcine mucin) and abiotic (glass and polystyrene) surfaces was studied, since they have been proposed as alternative models to study the adhesion of microorganisms in vitro [74]. It was observed that the attachment capabilities of selected probiotic strains differ between surfaces, which were in agreement with the results of Aleksandrzak-Piekarczyk et al. [32]. Based on the results obtained, it was concluded that the simplified model of adhesion assay would not bring results comparable to those acquired through the usage of an epithelial cell line, such as Caco-2, though they might provide a basic overview of biofilm formation properties.

## Conclusions

Based on the results obtained, it was concluded that selected *Lactobacillus* strains possess some desirable features which indicate their potential as probiotics. Five strains—*Lact. rhamnosus* ŁOCK 1087, *Lact. paracasei* ŁOCK 1091, *Lact. reuteri* ŁOCK 1092, *Lact. plantarum* ŁOCK 0860 and *Lact. pentosus* ŁOCK 1094—are suitable candidates for the development of new probiotic preparations or to serve as a component in synbiotics, along with a suitable prebiotic.

These probiotics are mostly isolated from monogastric animals which is why they can be recommended for use in pig or poultry feeding, after in vivo testing. The strains are considered able to survive gastrointestinal passage and to colonize the intestines as a result of their adhesion to epithelial cells and mucose. Moreover, the selected strains can prevent pathogenic bacteria colonization and thus to prevent the spread of infections. That is why the usage of the probiotic *Lactobacillus* strains could result in the improvement of safety and quality of meat or food products of animal origin.

## References

[1] Goldstein EJC, Tyrrell KL, Citron DM (2015). *Lactobacillus* species: taxonomic complexity and controversial susceptibilities. Clin Infect Dis.

[2] Riaz Rajoka MS, Mehwish HM, Siddiq M, Haobin Z, Zhu J, Yan L, Shao D, Xu X, Shi J (2017). Identification, characterization, and probiotic potential of *Lactobacillus rhamnosus* isolated from human milk. LWT - Food Sci Technol.

[3] Shi Y, Zhao J, Kellingray L, Zhang H, Narbad A, Zhai Q, Chen W (2018). *In vitro* and *in vivo* evaluation of *Lactobacillus* strains and comparative genomic analysis of *Lactobacillus plantarum* CGMCC12436 reveal candidates of colonise-related genes. Food Res Int.

[4] Archacka M, Białas W, Dembczyński R, Olejnik A, Sip A, Szymanowska D, Celińska E, Jankowski T, Olejnik A, Rogodzińska M (2019). Method of preservation and type of protective agent strongly influence probiotic properties of *Lactococcus lactis*: a complete process of probiotic preparation manufacture and use. Food Chem.

[5] FAO/WHO (2002) Guidelines for the evaluation of probiotics in food. Food and Agriculture Organization of the United Nations and World Health Organization Working Group Report. http://www.fao.org/3/a-a0512e.pdf. Accessed 02 December 2019

[6] Hill C, Guarner F, Reid G, Gibson GR, Merenstein DJ, Pot B, Morelli L, Canani RB, Flint HJ, Salminen S, Calder PC, Sanders ME (2014). Expert consensus document: the international scientific association for probiotics and prebiotics consensus statement on the scope and appropriate use of the term probiotic. Nat Rev Gastroenterol Hepatol.

[7] Sirichokchatchawan W, Pupa P, Praechansri P, Am-in N, Tanasupawat S, Sonthayanon P, Prapasarakul N (2018). Autochthonous lactic acid bacteria isolated from pig faeces in Thailand show probiotic properties and antibacterial activity against enteric pathogenic bacteria. Microb Pathog.

[8] de Melo Pereira GV, de Oliveira CB, Magalhães Júnior AI, Thomaz-Soccol V, Soccol CR (2018). How to select a probiotic? A review and update of methods and criteria. Biotechnol Adv.

[9] Maleki Kakelar H, Barzegari A, Hanifian S, Barar J, Omidi Y (2019). Isolation and molecular identification of *Lactobacillus* with probiotic potential from abomasums driven rennet. Food Chem.

[10] Iraporda C, Rubel IA, Manrique GD, Abraham AG (2019). Influence of inulin rich carbohydrates from Jerusalem artichoke (*Helianthus tuberosus* L.) tubers on probiotic properties of *Lactobacillus* strains. LWT - Food Sci Technol.

[11] Mohanty D, Panda S, Kumar S, Ray P (2019). *In vitro* evaluation of adherence and anti-infective property of probiotic *Lactobacillus plantarum* DM 69 against *Salmonella enterica*. Microb Pathog.

[12] Grigoryan S, Bazukyan I, Trchounian A (2018). Aggregation and adhesion activity of lactobacilli isolated from fermented products *in vitro* and *in vivo*: a potential probiotic strain. Probiotics Antimicrob Proteins.

[13] Suvarna S, Dsouza J, Ragavan ML, Das N (2018). Potential probiotic characterization and effect of encapsulation of probiotic yeast strains on survival in simulated gastrointestinal tract condition. Food Sci Biotechnol.

[14] Sakandar HA, Kubow S, Sadiq FA (2019). Isolation and *in-vitro* probiotic characterization of fructophilic lactic acid bacteria from Chinese fruits and flowers. LWT - Food Sci Technol.

[15] Armas F, Camperio C, Marianelli C (2017). *In vitro* assessment of the probiotic potential of *Lactococcus lactis* LMG 7930 against ruminant mastitis-causing pathogens. PLoS One.

[16] Iglesias MB, Abadias M, Anguera M, Sabata J, Viñas I (2017). Antagonistic effect of probiotic bacteria against foodborne pathogens on fresh-cut pear. LWT - Food Sci Technol.

[17] Chlebicz A, Śliżewska K (2018). Campylobacteriosis, salmonellosis, yersiniosis, and listeriosis as zoonotic foodborne diseases: a review. Int J Environ Res Public Health.

[18] Palaniyandi SA, Damodharan K, Suh JW, Yang SH (2017). *In vitro* characterization of *Lactobacillus plantarum* strains with inhibitory activity on enteropathogens for use as potential animal probiotics. Indian J Microbiol.

[19] Bacanlı M, Başaran N (2019). Importance of antibiotic residues in animal food. Food Chem Toxicol.

[20] Poloni V, Salvato L, Pereyra C, Oliveira A, Rosa C, Cavaglieri L, Keller KM (2017). Bakery by-products based feeds borne-*Saccharomyces cerevisiae* strains with probiotic and antimycotoxin effects plus antibiotic resistance properties for use in animal production. Food Chem Toxicol.

[21] Zuo ZH, Shang BJ, Shao YC, Li WY, Sun JS (2019). Screening of intestinal probiotics and the effects of feeding probiotics on the growth, immune, digestive enzyme activity and intestinal flora of *Litopenaeus vannamei*. Fish Shellfish Immunol.

[22] Guerra AF, Lemos Junior WJF, dos Santos GO, Andrighetto C, Gianomini A, Corich V, Luchese RH (2018). *Lactobacillus paracasei* probiotic properties and survivability under stress-induced by processing and storage of ice cream bar or ice-lolly. Ciência Rural.

[23] Zhou JS, Gopal PK, Gill HS (2001). Potential probiotic lactic acid bacteria *Lactobacillus rhamnosus* (HN001), *Lactobacillus acidophilus* (HN017) and *Bifidobacterium lactis* (HN019) do not degrade gastric mucin *in vitro*. Int J Food Microbiol.

[24] Strus M (1998). A new method for testing antagonistic activity of lactic acid bacteria (LAB) on selected pathogenic indicator bacteria. Med Dośw Mikrobiol.

[25] Zielińska D, Rzepkowska A, Radawska A, Zieliński K (2015). *In vitro* screening of selected probiotic properties of *Lactobacillus* strains isolated from traditional fermented cabbage and cucumber. Curr Microbiol.

[26] Halder D, Mandal M, Chatterjee S, Pal N, Mandal S (2017). Indigenous probiotic *Lactobacillus* isolates presenting antibiotic like activity against human pathogenic bacteria. Biomedicines.

[27] Rosenberg M, Gutnick D, Rosenberg E (1980). Adherence of bacteria to hydrocarbons: a simple method for measuring cell-surface hydrophobicity. FEMS Microbiol Lett.

[28] Chae MS, Schraft H, Hansen LT, Mackereth R (2006). Effects of physicochemical surface characteristics of *Listeria monocytogenes* strains on attachment to glass. Food Microbiol.

[29] Ben Taheur F, Kouidhi B, Fdhila K, Elabed H, Ben Slama R, Mahdouani K, Bakhrouf A, Chaieb K (2016). Anti-bacterial and anti-biofilm activity of probiotic bacteria against oral pathogens. Microb Pathog.

[30] Kos B, Šušković J, Vuković S, Sǐmpraga M, Frece J, Matošić S (2003). Adhesion and aggregation ability of probiotic strain *Lactobacillus acidophilus* M92. J Appl Microbiol.

[31] Handley PS, Harty DWS, Wyatt JE, Brown CR, Doran JP, Gibbs ACC (1987). A comparison of the adhesion, coaggregation and cell-surface hydrophobicity properties of fibrillar and fimbriate strains of *Streptococcus salivarius*. Microbiology.

[32] Aleksandrzak-Piekarczyk T, Koryszewska-Bagińska A, Grynberg M, Nowak A, Cukrowska B, Kozakova H, Bardowski J (2016). Genomic and functional characterization of the unusual pLOCK 0919 plasmid harboring the spaCBA pili cluster in *Lactobacillus casei* LOCK 0919. Genome Biol Evol.

[33] Tsai CC, Hsih HY, Chiu HH, Lai YY, Liu JH, Yu B, Tsen HY (2005). Antagonistic activity against *Salmonella* infection *in vitro* and *in vivo* for two *Lactobacillus* strains from swine and poultry. Int J Food Microbiol.

[34] Saidi N, Snoussi M, Usai D, Zanetti S, Bakhrouf A, Analyse L, De P, Biomediche S, Sperimentale M, Sassari U (2011). Adhesive properties of *Aeromonas hydrophila* strains isolated from Tunisian aquatic biotopes. African J Microbiol Res.

[35] Naqid IA, Owen JP, Maddison BC, Gardner DS, Foster N, Tchórzewska MA, La Ragione RM, Gough KC (2015). Prebiotic and probiotic agents enhance antibody-based immune responses to *Salmonella* Typhimurium infection in pigs. Anim Feed Sci Technol.

[36] Kritas SK, Di Gioia D, Biavati B (2018). Probiotics and prebiotics for the health of pigs and horses. Probiotics and prebiotics in animal health and food safety.

[37] Shokryazdan P, Faseleh Jahromi M, Liang JB, Kalavathy R, Sieo CC, Ho YW (2016). Safety assessment of two new *Lactobacillus* strains as probiotic for human using a rat model. PLoS One.

[38] Bhat MI, Singh VK, Sharma D, Kapila S, Kapila R (2019). Adherence capability and safety assessment of an indigenous probiotic strain *Lactobacillus rhamnosus* MTCC-5897. Microb Pathog.

[39] Adimpong DB, Nielsen DS, Sørensen KI, Derkx PMF, Jespersen L (2012). Genotypic characterization and safety assessment of lactic acid bacteria from indigenous African fermented food products. BMC Microbiol.

[40] Ficoseco CA, Mansilla FI, Maldonado NC, Miranda H, Fátima Nader-Macias ME, Vignolo GM (2018). Safety and growth optimization of lactic acid bacteria isolated from feedlot cattle for probiotic formula design. Front Microbiol.

[41] Nallala V, Jeevaratnam K (2018). Probiotic evaluation of antimicrobial *Lactobacillus plantarum* VJC38 isolated from the crop of broiler chicken. Microbiology.

[42] Abouloifa H, Rokni Y, Bellaouchi R, Ghabbour N, Karboune S, Brasca M, Ben Salah R, Chihib NE, Saalaoui E, Asehraou A (2019) Characterization of probiotic properties of antifungal *Lactobacillus* strains isolated from traditional fermenting green olives. Probiotics Antimicrob Proteins. 10.1007/s12602-019-09543-8 10.1007/s12602-019-09543-8 30929140

[43] Shehata MG, El Sohaimy SA, El-Sahn MA, Youssef MM (2016). Screening of isolated potential probiotic lactic acid bacteria for cholesterol lowering property and bile salt hydrolase activity. Ann Agric Sci.

[44] Owusu-Kwarteng J, Tano-Debrah K, Akabanda F, Jespersen L (2015). Technological properties and probiotic potential of *Lactobacillus fermentum* strains isolated from west African fermented millet dough applied microbiology. BMC Microbiol.

[45] Gharbi Y, Fhoula I, Ruas-Madiedo P, Afef N, Boudabous A, Gueimonde M, Ouzari HI (2019). *In-vitro* characterization of potentially probiotic *Lactobacillus* strains isolated from human microbiota: interaction with pathogenic bacteria and the enteric cell line HT29. Ann Microbiol.

[46] Jose NM, Bunt CR, Hussain MA (2015). Implications of antibiotic resistance in probiotics. Food Rev Int.

[47] Mejri L, Hassouna M (2016). Characterization and selection of *Lactobacillus plantarum* species isolated from dry fermented sausage reformulated with camel meat and hump fat. Appl Biol Chem.

[48] Casarotti SN, Carneiro BM, Todorov SD, Nero LA, Rahal P, Penna ALB (2017). *In vitro* assessment of safety and probiotic potential characteristics of *Lactobacillus* strains isolated from water buffalo mozzarella cheese. Ann Microbiol.

[49] Hyacinta M, Hana KS, Andrea B, Barbora Č (2015). Bile tolerance and its effect on antibiotic susceptibility of probiotic *Lactobacillus* candidates. Folia Microbiol (Praha).

[50] Rao KP, Chennappa G, Suraj U, Nagaraja H, Charith Raj AP, Sreenivasa MY (2015). Probiotic potential of *Lactobacillus* strains isolated from sorghum-based traditional fermented food. Probiotics Antimicrob Proteins.

[51] Sharma P, Tomar SK, Sangwan V, Goswami P, Singh R (2016). Antibiotic resistance of *Lactobacillus* sp. isolated from commercial probiotic preparations. J Food Saf.

[52] Dec M, Urban-Chmiel R, Stȩpień-Pyśniak D, Wernicki A (2017). Assessment of antibiotic susceptibility in *Lactobacillus* isolates from chickens. Gut Pathog.

[53] Devirgiliis C, Zinno P, Perozzi G (2013). Update on antibiotic resistance in foodborne *Lactobacillus* and *Lactococcus* species. Front Microbiol.

[54] Mallappa RH, Singh DK, Rokana N, Pradhan D, Batish VK, Grover S (2019). Screening and selection of probiotic *Lactobacillus* strains of Indian gut origin based on assessment of desired probiotic attributes combined with principal component and heatmap analysis. LWT - Food Sci Technol.

[55] Dec M, Nowaczek A, Urban-Chmiel R, Stepien-Pysniak D, Wernicki A (2018). Probiotic potential of *Lactobacillus* isolates of chicken origin with anti-*Campylobacter* activity. J Vet Med Sci.

[56] Klose V, Bayer K, Bruckbeck R, Schatzmayr G, Loibner AP (2010). *In vitro* antagonistic activities of animal intestinal strains against swine-associated pathogens. Vet Microbiol.

[57] Shokryazdan P, Sieo CC, Kalavathy R, Liang JB, Alitheen NB, Faseleh Jahromi M, Ho YW (2014). Probiotic potential of *Lactobacillus* strains with antimicrobial activity against some human pathogenic strains. Biomed Res Int.

[58] Choi AR, Patra JK, Kim WJ, Kang SS (2018). Antagonistic activities and probiotic potential of lactic acid bacteria derived from a plant-based fermented food. Front Microbiol.

[59] Pessoa WFB, Melgaço ACC, De Almeida ME, Ramos LP, Rezende RP, Romano CC (2017). *In vitro* activity of lactobacilli with probiotic potential isolated from cocoa fermentation against *Gardnerella vaginalis*. Biomed Res Int.

[60] Li Q, Liu X, Zhou J, Wang Y (2015). Aggregation and adhesion abilities of 18 lactic acid bacteria strains isolated from traditional fermented food. Int J Agric Policy Res.

[61] Ait Seddik H, Bendali F, Cudennec B, Drider D (2017). Anti-pathogenic and probiotic attributes of *Lactobacillus salivarius* and *Lactobacillus plantarum* strains isolated from feces of Algerian infants and adults. Res Microbiol.

[62] Saini K, Tomar SK (2017). *In vitro* evaluation of probiotic potential of *Lactobacillus* cultures of human origin capable of selenium bioaccumulation. LWT - Food Sci Technol.

[63] do Carmo MS, FMF N, Arruda MO, da Silva Costa ÊP, MRQ B, Monteiro AS, TAF F, Fernandes ES, Girón JA, Monteiro-Neto V (2016). *Lactobacillus fermentum* ATCC 23271 displays *in vitro* inhibitory activities against *Candida* spp. Front Microbiol.

[64] de Souza BMS, Borgonovi TF, Casarotti SN, Todorov SD, Penna ALB (2018). *Lactobacillus casei* and *Lactobacillus fermentum* strains isolated from mozzarella cheese: probiotic potential, safety, acidifying kinetic parameters and viability under gastrointestinal tract conditions. Probiotics Antimicrob Proteins.

[65] Kaktcham PM, Temgoua JB, Zambou FN, Diaz-Ruiz G, Wacher C, de Pérez-Chabela ML (2018). *In vitro* evaluation of the probiotic and safety properties of bacteriocinogenic and non-bacteriocinogenic lactic acid bacteria from the intestines of nile tilapia and common carp for their use as probiotics in aquaculture. Probiotics Antimicrob Proteins.

[66] Tareb R, Bernardeau M, Gueguen M, Vernoux JP (2013). *In vitro* characterization of aggregation and adhesion properties of viable and heat-killed forms of two probiotic *Lactobacillus* strains and interaction with foodborne zoonotic bacteria, especially *Campylobacter jejuni*. J Med Microbiol.

[67] Campana R, Van Hemert S, Baffone W (2017). Strain-specific probiotic properties of lactic acid bacteria and their interference with human intestinal pathogens invasion. Gut Pathog.

[68] Gómez NC, Ramiro JMP, Quecan BXV, de Melo Franco BDG (2016). Use of potential probiotic lactic acid bacteria (LAB) biofilms for the control of *Listeria monocytogenes, Salmonella Typhimuriu*m, and *Escherichia coli* O157: H7 biofilms formation. Front Microbiol.

[69] Feng J, Liu P, Yang X, Zhao X (2015). Screening of immunomodulatory and adhesive *Lactobacillus* with antagonistic activities against *Salmonella* from fermented vegetables. World J Microbiol Biotechnol.

[70] Archer AC, Kurrey NK, Halami PM (2018). *In vitro* adhesion and anti-inflammatory properties of native *Lactobacillus fermentum* and *Lactobacillus delbrueckii* spp. J Appl Microbiol.

[71] Jessie Lau LY, Chye FY (2018). Antagonistic effects of *Lactobacillus plantarum* 0612 on the adhesion of selected foodborne enteropathogens in various colonic environments. Food Control.

[72] Ramos CL, Thorsen L, Schwan RF, Jespersen L (2013). Strain-specific probiotics properties of *Lactobacillus fermentum, Lactobacillus plantarum* and *Lactobacillus brevis* isolates from Brazilian food products. Food Microbiol.

[73] Kim JH, Baik SH (2019). Probiotic properties of *Lactobacillus* strains with high cinnamoyl esterase activity isolated from jeot-gal, a high-salt fermented seafood. Ann Microbiol.

[74] Grajek W, Olejnik A, Sip A (2005). Probiotics, prebiotics and antioxidants as functional foods. Acta Biochim Pol.

